# Wide Local Excision for Dermatofibrosarcoma Protuberans: A Single-Center Series of 90 Patients

**DOI:** 10.1155/2015/642549

**Published:** 2015-11-25

**Authors:** Byung Jun Kim, Hyeonwoo Kim, Ung Sik Jin, Kyung Won Minn, Hak Chang

**Affiliations:** Department of Plastic Surgery, College of Medicine, Seoul National University, 101 Daehak-ro, Jongno-gu, Seoul 110-744, Republic of Korea

## Abstract

*Background*. Dermatofibrosarcoma protuberans (DFSP), a rare low-grade sarcoma of fibroblast origin, tends to extend in a finger-like fashion beyond macroscopic tumor margins. Therefore, incomplete removal and subsequent recurrence are common. This study aimed to determine the efficacy of wide local excision (WLE) for controlling local recurrence of DFSP.* Methods*. The medical records of 90 DFSP patients who received WLE at our hospital between June 1992 and January 2015 were retrospectively reviewed. WLE was conducted including a 3 cm (range, 1 to 5 cm) safety margin according to tumor size, location, and recurrence status. Clinical and tumor characteristics and surgical methods were evaluated for risk factor analysis and local recurrence-free survival.* Results*. DFSP occurred most often in patients in their 30s (30%) and on the trunk (51.1%). Five patients (5.5%) experienced local recurrence during the 43.4-month follow-up period. Recurrence was found at a mean of 10.8 months after WLE. Although no factors were significantly associated with recurrence, recurrences were more frequent in head and neck. Recurrence-free survival was 87% in 6 years and 77% in 7 years.* Conclusions*. WLE with adequate lateral and deep margins can effectively control local recurrence rate and is a simple and effective method to treat DFSP.

## 1. Introduction

Dermatofibrosarcoma protuberans (DFSP) is a rare locally aggressive mesenchymal tumor of fibroblast origin; it accounts for only 1% of all soft tissue sarcomas and <0.1% of all malignancies [1]. The incidence of DFSP is approximately 3–5 cases per million persons [2–4]. It usually occurs in young adults (aged between 20 and 40 years), and there is no definite predominance considering the sex of the patient [5].

DFSP can occur in any part of the body, but the trunk is the most common area involved, followed by the extremities and the head and neck [6]. DFSP grows slowly, similar to nodules that appear as hypertrophic scars or benign soft tissue tumors without any definite symptoms. Delays in diagnosis are common because of the benign appearance and its rarity.

DFSP rarely metastasizes to the regional lymph nodes or distant organs, with the possibility of metastasis being <5% [7, 8]. However, DFSP shows locally aggressive behavior and the local recurrence rate is between 0% and 60% [9]. The tumor infiltrates the surrounding dermis and subcutaneous tissue as a pseudopod; therefore, incomplete removal is common owing to its irregular shape. Hence, the local recurrence rate is high. Deciding the proper surgical margin for complete resection is challenging. Therefore, many studies have shown the superiority of Mohs micrographic surgery (MMS) compared to wide local excision (WLE) while considering the local control of DFSP [10–13]. MMS has advantages in controlling the tumor burden microscopically, but it is laborious, technically demanding, expensive, and time-consuming, which are major disadvantages [14].

Therefore, the purpose of this study was to determine the efficacy of WLE with a proper resection margin by using a large amount of clinical data on DFSP at a single center in Korea.

## 2. Materials and Methods

The medical records of patients with DFSP confirmed on histologic analysis and who were treated at our hospital between June 1992 and January 2015 were retrospectively reviewed under the approval of the institutional review board (IRB number 1508-157-699). The patients who were diagnosed with DFSP but did not undergo WLE at our hospital were excluded from this study. We examined the medical records to obtain medical information of patients with DFSP, including the clinical and tumor characteristics as well as the surgical methods and outcomes. This information piece was used to investigate the clinical course of DFSP and the prognostic factors associated with recurrence after WLE.

### 2.1. Treatment Protocol

Most of the patients with DFSP were referred to our clinic from primary clinics, for definite surgical treatment after incomplete excision or owing to recurrent lesions. Patients were categorized as primary versus recurrence cases. The patients were defined as having primary DFSP when histology confirmed DFSP and WLE was performed at our hospital within 3 months after diagnosis, irrespective of the place where diagnosis was confirmed. The patients were defined as having recurrence when the DFSP mass was surgically excised using excisional biopsy or WLE methods, but new lesions were found and confirmed to be DFSP.

Preoperative diagnostic workups included a general laboratory checkup, chest radiography, magnetic resonance imaging (MRI) of the primary lesion, and positron emission tomography (PET) to rule out the involvement of the regional lymph nodes or metastasis to distant organs. Bone scintigraphy was also performed when bony tissue was likely to be involved, as observed on radiologic imaging. Biopsy examination slides that had been obtained at other hospitals were reconfirmed by the pathologists at our hospital.

**Figure 1:**
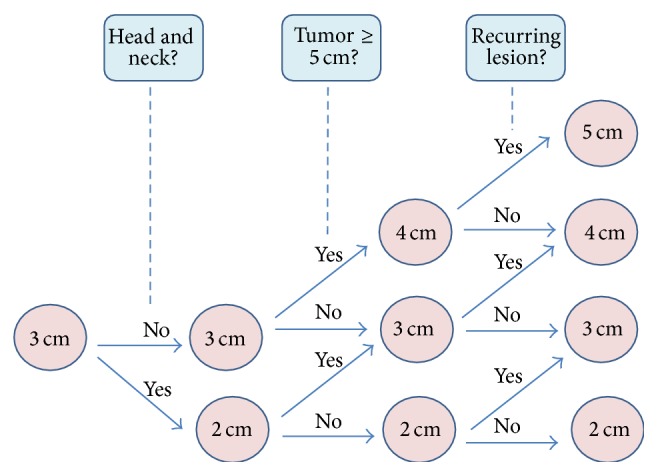
The flow sheet shows how to determine resection margins. The area of involvement, tumor size, and recurrence status affect the determination of the resection margin.

**Figure 2:**
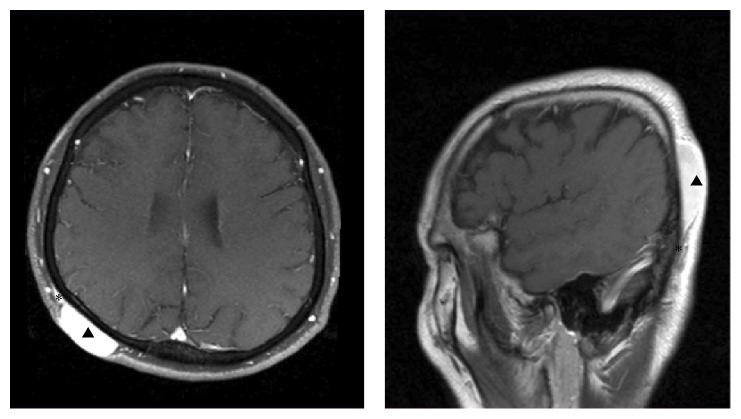
MRI image showing the subcutaneous extension of DFSP. These images help to determine the outline of DFSP. Arrow heads present primary lesion of DFSP in scalp. Asterisks present subcutaneous extension beyond the macroscopic tumor margin. MRI: magnetic resonance imaging, DFSP: dermatofibrosarcoma protuberans.

WLE was performed in all patients, with the patient under general anesthesia. The lateral margins were decided based on the size of tumors, the location of DFSP, recurrence status, and the pattern of spreading on MRI imaging. The standard resection margin was 3 cm. Another 1 cm was added for masses exceeding 5 cm (i.e., huge masses) or for recurrent lesions. More conservative resection margins were employed for smaller lesions or for those located in the head and neck in order to prevent aesthetic and functional impairment (Figure 1). MRI helped us to determine the area of primary lesions. Subcutaneous extension of DFSP, which is hard to be identified on physical examinations, can be easily discovered using MRI (Figure 2). Deep resection margins routinely included the muscle fascia except for primary cases with smaller tumors. Intraoperative frozen biopsy was usually conducted to identify the remnant tumor burden after excision with lateral and deep margins. If frozen biopsy revealed positive resection margins, additional WLE was performed from the point at which positive results were obtained.

**Figure 3:**
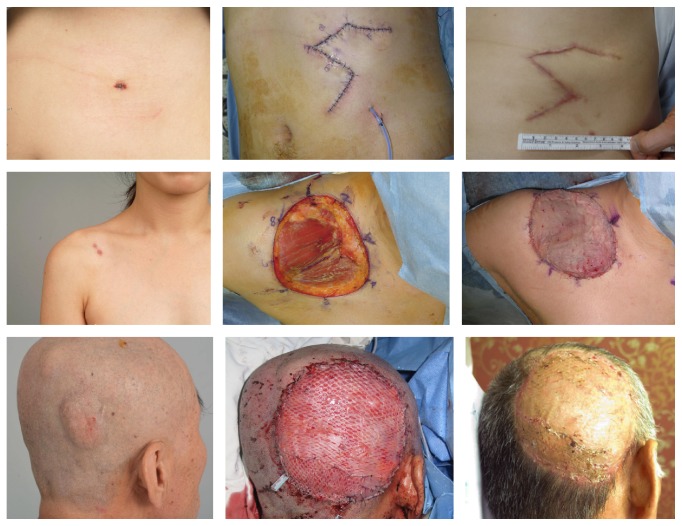
Intraoperative photos show WLE of DFSP and reconstruction with a local flap, skin graft, and free flap, respectively. WLE: wide local excision, DFSP: dermatofibrosarcoma protuberans.

Immediate reconstruction was performed after WLE was completed, and frozen biopsy revealed negative resection margins. Relatively small defects in abdomen can be repaired by primary closure without difficulty. In larger defects that do not permit primary closure, skin grafts or local flaps were employed. Free flaps can be another option for large defects or aesthetically important areas, such as the head and neck to avoid depressive pigmented scars from skin grafts (Figure 3). In case of positive resection margins on permanent biopsy, reoperation was performed whenever possible to obtain a clear margin. Adjuvant treatment including radiation or chemotherapy was performed only for huge masses, recurrent lesions, or inoperable cases.

Postoperative surveillance at the primary site and the regional lymph nodes was performed in 3 and 6 months by using physical examinations, chest radiography, and ultrasonography. MRI or PET was performed in highly suspicious cases of recurrence or metastasis. From 1 year after surgery, annual checkups were performed until 5 years by using the same methods.

### 2.2. Statistical Analysis

The Kaplan-Meier method was used to evaluate the status of recurrence. Univariate and multivariate analyses were performed using the Cox regression test to identify risk factors that were associated with recurrence. Statistical analysis was performed using the SAS program (version 9.3; SAS Institute Inc., USA). *P*-values less than 0.05 were considered statistically significant.

## 3. Results

### 3.1. Clinical Characteristics

**Figure 4:**
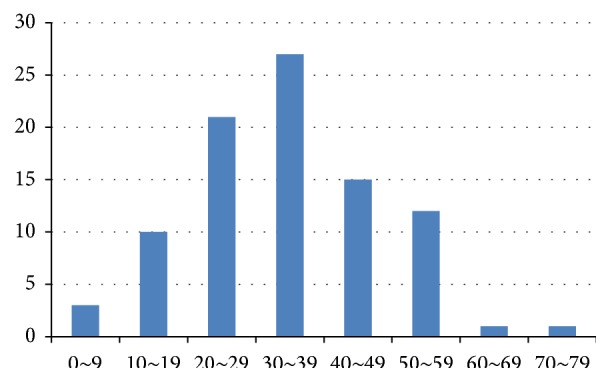
Age distribution presents a peak in patients in their 30s with a normal distribution pattern.

**Figure 5:**
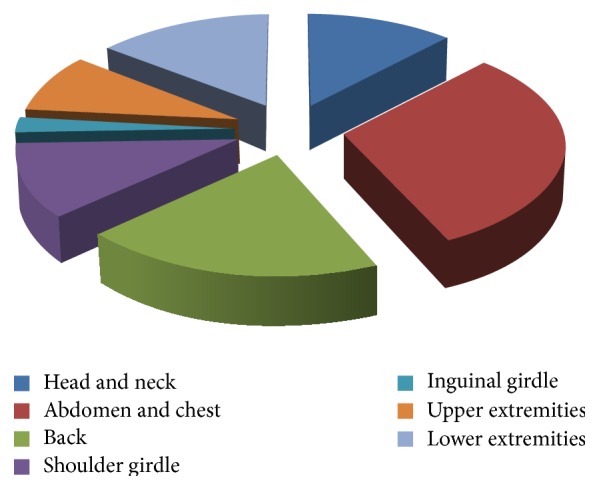
Circular graph shows distribution of location where DFSP developed. DFSP: dermatofibrosarcoma protuberans.

Of 90 subjects included in the present study, 53 (58.9%) were men and 37 (41.1%) were women. DFSP mostly occurred between the age of 20 and 50 years, and the mean age at surgery was 34.6 years (range, 0–78 years). The age distribution showed a normal distribution pattern with a peak for patients in their 30s (Figure 4). The mean duration from onset to surgery was 56.4 months. The trunk (51.1%) was the most common site involved, followed by the upper extremities (20.0%), lower extremities (16.6%), and head and neck (12.2%) (Figure 5). Most tumors did not exceed 30 mm; 5 tumors were larger than 50 mm. Seventy-seven (85.6%) patients visited our clinic with de novo tumors or for definitive treatment after incomplete excision from another hospital. Thirteen (14.4%) cases of recurrence were initially misdiagnosed as benign lesions; these patients underwent excision at another clinic without any histological diagnosis (Table 1).

### 3.2. Treatment Characteristics

All patients underwent WLE with adequate margins. The mean lateral resection margin was 2.94 cm (range, 1–5 cm). In 58 patients (64.4%), the tumor was resected including the deep fascia in order to obtain adequate deep margins. Permanent biopsy revealed positive results in 4 patients (4.4%). Additional WLE was performed in all of these patients to remove the tumor cells completely. The reconstruction methods used were skin grafts in 38.9% of the patients, primary closure in 27.8%, local flaps in 23.3%, and free flaps in 10.0%. There were no major complications after surgery, such as flap failure. Minor complications such as partial graft loss, local flap congestion, or wound dehiscence were treated with conservative management (Table 2). Six patients underwent postoperative radiotherapy after tumor excision; 3 of them had huge tumors while the other 3 had a history of repeated recurrence. Two patients received chemoradiotherapy; 1 patient had a huge tumor in the forehead that had recurred repeatedly despite WLE, and the other patient had DFSP in her upper lip that could not be excised with adequate margins. The mean radiation dose was 57.0 Gy (range, 50.0–63.0 Gy).

### 3.3. Prognostic Factors

**Figure 6:**
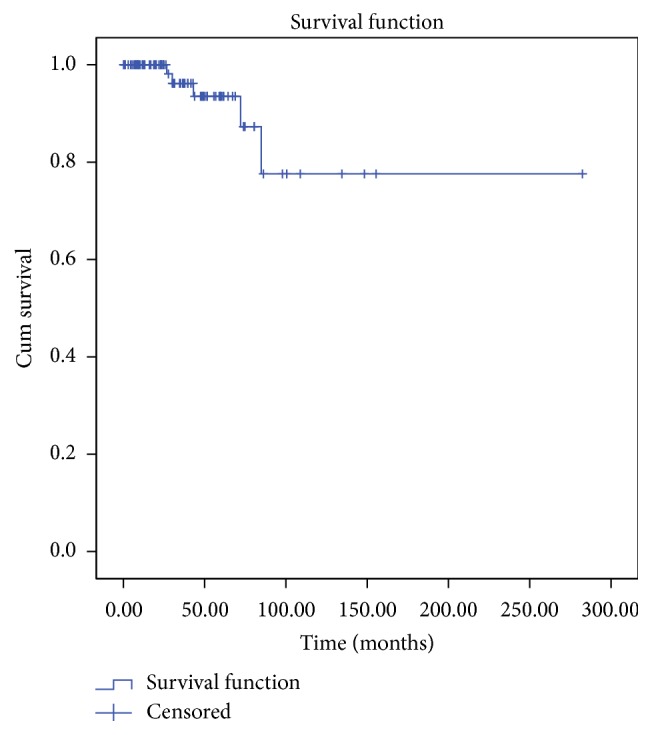
Survival curve represents the recurrence-free survival. Median follow-up was 3.6 years. The 6-year recurrence-free survival and 7-year recurrence-free survival were 87% and 77%, respectively.

The mean follow-up period was 43.4 months (range, 0.2–282.4 months). Local recurrence was found in 5 patients (5.5%); the mean period between surgery and recurrence in these patients was 10.8 months (range, 3–21 months). All recurrences occurred in the head and neck (2 patients), shoulder (1 patient), lower limb (1 patient), and inguinal area (1 patient); that is, none of them occurred on the trunk, which is usually the most common location. Additional WLE with skin grafts was performed in these patients, and adjuvant radiation therapy was performed in 2 patients. There were no cases of metastasis to the regional lymph nodes or distant organs. The Cox regression test did not identify any significant risk factors among the clinical characteristics and surgical methods that were related to local recurrence on both univariate and multivariate analyses. Kaplan-Meier survival analysis showed that the recurrence-free survival was 87% in 6 years and 77% in 7 years (Figure 6).

## 4. Discussion

In the present study, DFSP occurred most often in patients in their 30s (30%) and on the trunk (51.1%) without definite predominance in sex distribution. This corresponds well with the observation of previous articles [5, 19, 21]. Our data revealed relatively low risk of local recurrence rate (5.5%) after WLE, which is compatible with those observed after MMS [24, 25].

MMS is one of the most prevalent methods to treat skin cancer or sarcoma. MMS has shown a low risk of local recurrence as well as reduced positive resection margins. Previous studies have presented the superior outcomes of MMS to WLE regarding the local recurrence rate, although no randomized controlled studies have been performed [10, 12, 13, 26, 27]. These studies showed recurrence rates of 0% to 6.6% after MMS, whereas the rates increased from 11.0% to 35% after WLE. Relatively high local recurrence rate after WLE is attributable to incomplete resection margin of DFSP. Akram et al. reviewed articles regarding recurrence rates after WLE performed during 2000–2012 [28]. The pooled recurrence rate was 8.5% in 1432 patients, and the lower recurrence rate was related to wider excision. A review article by Pallure et al. showed that WLE with less than a 3 cm resection margin resulted in an increased recurrence rate [29].

We used 3 cm lateral resection margins but reduced them to 2 cm or less for smaller lesions in the head and neck while considering aesthetics. In recurrence cases or in case of huge tumors, an additional 1 cm resection margin was obtained to prevent the risk of further recurrence. Our protocol in determining the lateral resection margin is compatible with that used in previous studies that evaluated the resection margins and the prognosis of DFSP (Table 3). We also removed the deep fascia in most of the cases (64.4%) except for the primary cases with smaller tumors. Many authors suggested the importance of deep margin control while describing clinical cases with deep tissue invasion [24, 30, 31]. Fields et al. [32] recommended removing the deep fascia to completely eliminate vertical infiltrating cells based on the fact that tumor depth is associated with disease-free survival. According to Loghdey et al., achieving sufficient deep margins is important because of the nonconcentric extension pattern of DFSP and the limitation of standard vertical sections on histology [14].

In our study, 5 of 90 patients (5.5%) showed recurrence, which is relatively lower than that observed in previous articles. Establishing proper resection margins using MRI, intraoperative frozen biopsy, and active additional treatment according to the result of biopsy accounts for lower recurrence rate. MRI is effective in determining the outline of DFSP that grows beyond the macroscopic tumor margin [33–35]. MRI can give us visual clues regarding the tumor extension area, tumor depth, and the relationship with adjacent tissues. In addition, intraoperative frozen biopsy is a useful tool to minimize positive resection margin; it is also cost-effective [36]. In our study, 4 cases (4.4%) showed positive margins on frozen biopsy; therefore, additional WLE was performed. Permanent biopsy revealed negative results in all of these cases.

Other prognostic factors include old age, DFSP with a high-grade fibrosarcomatous component (FS-DFSP), recurrence, the involved site, increased mitosis, and positive microscopic margins [5, 26, 37, 38]. In our study, the head and neck and the extremities were associated with high rates of local recurrence, although the result was not significant, possibly because of the relatively thin subcutaneous layer in these locations compared to the trunk. Relative conservative treatment in the head and neck could be another cause of recurrence. This corresponds with the observation made by Paradisi et al. [10]. Meticulous excision and close follow-ups should be performed when WLE is performed in these areas. Well-designed MMS could be a better surgical option in treating DFSP of head and neck lesion, because MMS allows greater preservation of normal healthy tissue and better cosmetic results can be expected [39]. Future studies are required to determine the relationship between the tumor location and local recurrence. Our study failed to identify risk factors associated with local recurrence owing to the small number of patients with recurrence for statistical analysis. In addition, we modified the resection margin according to the location, tumor size, and recurrence status; therefore, it was difficult to discover factors related to recurrence on univariate analysis.

We restrictively performed adjuvant therapy for huge tumors, of frequent recurrence, or when significant morbidity is anticipated following WLE in functional and aesthetic aspects. The National Comprehensive Cancer Network guidelines also recommend limited use of adjuvant radiotherapy or chemotherapy [9]. Protein tyrosine kinase inhibitors (e.g., imatinib mesylate) can be efficaciously used for treating unresectable or metastatic DFSP in patients with translocation between chromosomes 17 and 22 (*t*(17:22)). Imatinib mesylate was approved by the FDA for the treatment of unresectable, metastatic DFSP in adults [40]. Although not used in our study, imatinib mesylate can be effectively used for uncontrolled metastatic DFSP in patients with positive cytogenetic study.

This study presents the findings from a large number of cases of DFSP in a single center in Korea. We focused on the detailed surgical protocol and its prognosis in the long term. WLE is a simple and effective method for treating DFSP, and local control can be improved comparable to MMS if adequate resection margins are established. However, this study had the limitation of being a retrospective study without a control group. Most of the patients were referred to our hospital by primary physicians after simple excision; therefore, it was difficult to obtain accurate information about the characteristics of the primary tumor and the histologic results.

In conclusion, the results of this study show the epidemiology and clinical characteristics in Korean patients with DFSP, which is compatible with those of previous studies. WLE with adequate lateral and deep margins can be effectively used to control local recurrence.

## Figures and Tables

**Table 1:** Clinical characteristics of 90 patients with DFSP.

Factors	Number of patients			Percentage (%)
Sex				
Male		53		58.9
Female		37		41.1

	Male	Female	Total	

Age				
0–9	3	0	3	3.3
10–19	4	6	10	11.1
20–29	15	6	21	23.3
30–39	15	12	27	30.0
40–49	9	6	15	16.7
50–59	6	6	12	13.3
60–69	1	0	1	1.1
70–79	0	1	1	1.1

	Male	Female	Total	

Location				
Head/neck	5	6	11	12.2
Abdomen/chest	19	9	28	31.1
Back	9	9	18	20.0
Shoulder girdle	6	4	10	11.1
Inguinal girdle	1	1	2	2.2
Upper extremities	7	1	8	8.9
Lower extremities	6	7	13	14.4

Tumor size				
<30 mm			58	64.4
30–50 mm			19	21.1
>50 mm			5	5.6
Unknown			8	8.9

Clinical presentation				
Primary			77	85.6
Recurred			13	14.4

Number of recurrences				
1			7	7.8
2			4	4.4
≥3			2	2.2

Duration prior to surgery (onset)				
1 year			25	27.8
2 years			9	10.0
3 years			9	10.0
>3 years			29	32.2
Unknown			18	20.0

DFSP: dermatofibrosarcoma protuberans.

**Table 2:** Surgical characteristics of 90 patients with DFSP.

Factors	Number of patients	Percentage (%)
Resection margin		
<30 mm	23	25.6
30–50 mm	51	56.7
≥50 mm	6	6.7
Unknown	10	11.1
Resection depth		
Subcutaneous	13	14.4
Deep fascia	58	64.4
Unknown	19	21.1
Surgical margin in biopsy		
Negative	85	94.4
Positive	4	4.4
Unknown	1	1.1
Reconstructive methods		
Primary closure	25	27.8
Skin graft	35	38.9
Local flap	21	23.3
Free flap	9	10.0

DFSP: dermatofibrosarcoma protuberans.

**Table 3:** Review of the literature regarding the clinical characteristics and surgical outcome in the treatment of DFSP.

Authors	Publishing year	Study design	Number of patients (male/female)	Age (years)	Resection margin (cm)	Adjuvant treatment	Overall recurrence rate (%)	Recurrence period (months)	Follow-up period (years)
Smola et al. [15]	1991	Retrospective	20	NR	1.0–5.0	NR	30.0	NR	8.8
Rutgers et al. [16]	1992	Retrospective	11	NR	>2.0	NR	0.0	NR	13.2 (2–28)
Stojadinovic et al. [17]	2001	Retrospective	14 (6/8)	37 (26–73)	≥2.0 (12), <2.0 (2)	NR	7.0	NR	8.4 (1.0–15.6)
Chang et al. [18]	2004	Retrospective	60 (23/37)	36 (10–70)	3.1 (1.0–6.0)	RT (3)	16.7	38.0 (1–100)	4.9
Monnier et al. [4]	2006	Retrospective	66 (36/30)	32.8 (0–67)	<0.9–5.0	NR	27.0	31.8	9.6
Popov et al. [19]	2007	Prospective	40 (22/18)	45	3.1	NR	0.0	NR	3.3
Paradisi et al. [10]	2008	Retrospective	38 (16/22)	44 (10–83)	2.0–5.0	NR	13.0	NR	4.8 (2–10)
Heuvel et al.[20]	2010	Retrospective	38 (19/11)	38 (8–77)	2.0–3.0	RT (8)	10.5	NR (81, 11)	7.4 (1.0–21.8)
Farma et al. [21]	2010	Retrospective	204 (76/128)	41 (1–84)	2.0 (0.5–3.0)	RT (9)	1.0	48.5 (13–84)	5.3 (0.1–17.5)
Cai et al. [22]	2012	Retrospective	223	38 (11–80)	>1.5	RT (34)	8.5	18.5 (3–129)	7.0 (0.6–23.8)
Hamid et al. [23]	2013	Retrospective	45 (30/15)	38.4 (7–65)	2.0–3.0	NR	22.2	32.0	5.7 (0.5–13)
Goldberg et al. [11]	2015	Retrospective	25 (8/17)	46.1 (12–84)	2.8	RT (1)	0.0	NR	9.0
Our study		Retrospective	90 (53/37)	34.6 (0–78)	3.0 (1.0–5.0)	RT (6) RT + C (2)	5.5	10.0 (5–14)	3.6 (0.1–23.5)

DFSP: dermatofibrosarcoma protuberans, RT: radiation, and NR: not recorded.
